# The effect of the papillary renal cell carcinoma subtype on oncological outcomes

**DOI:** 10.1038/s41598-020-78174-9

**Published:** 2020-12-03

**Authors:** Honghong Pan, Liefu Ye, Qingguo Zhu, Zesong Yang, Minxiong Hu

**Affiliations:** 1 Shengli Clinical Medical College, Fujian Medical University, Fuzhou, 350001 China; 2 Department of Urology, Fujian Provincial Hospital, Fuzhou, 350001 China; 3 Department of Urology, Jinshan Branch of Fujian Provincial Hospital, Fuzhou, 350028 China

**Keywords:** Cancer, Cancer, Kidney diseases, Urogenital diseases

## Abstract

The study aimed to compare the clinicopathological features and prognosis between type I and type II papillary renal cell carcinoma (PRCC) and to investigate whether the subtypes of PRCC would affect oncological outcomes. A total of 102 patients with PRCC were recruited, of which 42 were type I PRCC and 60 type II. The clinicopathological features and oncologic outcomes of the patients were evaluated. The type II cases had a higher WHO/ISUP grading (*P* < 0.001), T (*P* = 0.003), N (*P* = 0.010) stage and stage grouping (*P* = 0.011) than the type I. During a median follow-up period of 61.4 months, 1-year cancer specific survival (CSS) of the type I was 100%, 5-year CSS was 95.2%, the 1-year CSS of the type II was 96.2%, and 5-year CSS was 75.7%. The univariate analysis showed that subtype, symptoms, TNM, stage grouping, WHO/ISUP grading and surgical methods appeared to affect prognosis of the patients with PRCC. However, multivariate analysis revealed that only stage grouping was the independent risk factor. After the stage grouping factor was adjusted for the analysis, there were no statistically significant differences in CSS (*P* = 0.214) and PFS (*P* = 0.190) between the localized type I and type II PRCC groups. Compared with type I PRCC, type II had higher pathological T, N stage and WHO/ISUP grading. However, it was the Stage grouping that made a great difference to oncological outcomes, rather than the subtype of PRCC.

## Introduction

With the improvement of people's health awareness and the rapid development of imaging techniques, detection rate of renal cell carcinoma has increased year by year. Papillary renal cell carcinoma (PRCC) is a primary malignant tumor of the renal tubular epithelium first reported by Mancilla-Jimenez^1^ in 1976. Since then, it has become an independent and clinicopathological type of renal cell carcinoma. PRCC accounts for 15 to 20% of renal cell carcinoma cases. As the second most common renal cell carcinoma, PRCC is with its own characteristics which is constantly being redefined clinically.

Traditionally, PRCC may be classified into type I (pale cytoplasm, small-cell) and type II (eosinophilic cytoplasm, large cell). Previous studies had shown that the two types have marked differences in imaging and prognosis^2,3^. Type II of PRCC was found to have a higher stage, a higher nuclear grade and worse outcomes than type I^2^. But more recently a number of studies have reported that the subtypes of PRCC had no effect on the oncologic prognosis postoperatively in Europe and America^4–6^. Therefore whether the subtype of PRCC affects the prognosis has remained controversial. In addition, almost all researches were conducted in Europe and America. Few researches were carried out in Asia areas.

The present study was to investigate clinicopathologic differences between the two subtypes of PRCC, type I and type II, in Chinese patients. An additional objective was to determine whether the subtypes would affect the prognosis.

## Results

### Information

Of the 102 PRCC patients included in the study, 76 (74.5%) of patients were male and other 26 (25.5%) were female; the male to female ratio was 2.9:1 (76:26). The subjects aged between 9 to 93 years old with an average age of 53.8 ± 15.0 years old. 64.7% of patients were asymptomatic, who were found accidentally by health examination or with other diseases. Among the remaining cases, most patients suffered flank pain (72.2%) and hematuria (22.2%). The follow-up time ended as of September 30, 2020. Of the 102 cases, 5 were lost after operation, while the remaining 97 cases were followed up with a median follow-up time of 61.4 (3–153) months.

### Comparison of characteristics between type I and type II PRCC

As shown in Table 1, 42 patients had type I PRCC and 60 patients had type II PRCC. The average age of type I was 53.2 years old, which was similar to type II (54.2, *P* = 0.766). In terms of symptoms, whether in type I or type II group, most of patients were asymptomatic. Compared with type I patients, more type II patients presented symptoms. Yet there were no statistical differences between them (*P* = 0.443). In addition, hematuria and flank pain were common symptoms in both groups, while other symptoms included weight loss and fever. As for pathological stage (TNM), there was not a T4 -Stage patient in this study. Most type I patients were in T1 and T2 stage. The majority of patients in T3 and T4 stage were type II PRCC. There existed significant differences between the two groups (*P* = 0.003). Additionally, patients with type II PRCC were more likely to develop lymph node metastasis than patients with type I (15% vs. 0%, *P* = 0.010). However, the probability of distant metastasis in the type II group were similar to that in type I group (4.8% vs. 5.0%, *P* = 1.000). Patients with type II PRCC had more advanced stage grouping (*P* = 0.011) and larger tumor diameter (*P* < 0.001) than that in type I. Concerning WHO/ISUP grading, the number of type II patients in high-grade (Grade 3–4) accounted for 46.7%, while type I patients only accounted for 9.5% (*P* < 0.001). Furthermore, there were no Grade 4 patients. As for treatment methods, there was no significant difference between the two subtypes (*P* = 0.930).

**Table 1:** Clinicopathologic characteristics of patients with PRCC.

	Type I (n = 42)	Type II (n = 60)	*P*
**Age** (years)	53.2 ± 15.1	54.2 ± 15.0	0.766^a^
**Gender**			0.431^b^
Male	33 (79%)	43 (72%)	
Female	9 (21%)	17 (28%)	
**Initial symptoms**			0.443^b^
Presenting Symptoms	13 (31%)	23 (38%)	
No symptoms	29 (69%)	37 (62%)	
**Treatment method**			0.930^c^
Radical nephrectomy	23 (55%)	34 (57%)	
Nephron sparing surgery	18 (43%)	24 (40%)	
Radiofrequency ablation	1 (2%)	2 (3%)	
**Tumor size** (cm)	3.1 (2–5)	3.6 (3–5.9)	0.176^d^
**Pathologic T stage**			0.003^c^
1	35 (83%)	45 (75%)	
2	7 (17%)	4 (7%)	
3	0 (0%)	11 (18%)	
**Pathologic N stage**			0.010^c^
0	42 (100%)	51 (85%)	
1	0 (0%)	9 (15%)	
**Pathologic M stage**			1.000^c^
0	40 (95%)	57 (95%)	
1	2 (5%)	3 (5%)	
**Stage grouping**			0.011^c^
Stage I	35 (83%)	42 (70%)	
Stage II	5 (12%)	4 (7%)	
Stage III	0 (0%)	11 (18%)	
Stage IV	2 (5%)	3 (5%)	
**WHO/ISUP grading**			< 0.001^e^
1	5 (13%)	1 (2%)	
2	31 (77%)	29 (50%)	
3	4 (10%)	27 (46%)	
4	0 (0%)	1 (2%)	

^a^Two- sample test; ^b^Pearsonχ^2^ Test; ^c^Fisher's Exact Probability Method; ^d^Mann–Whitney U Test; ^e^Wilcoxon Test.

### Comparison of survival prognosis between type I and type II PRCC

In this study, 5 cases lost follow-up (1 case of type I and 4 of type II) and were treated as censored. The average follow-up time was 61.4 months. To the end of follow-up, a total of 12 death occurred.

**Figure 1:**
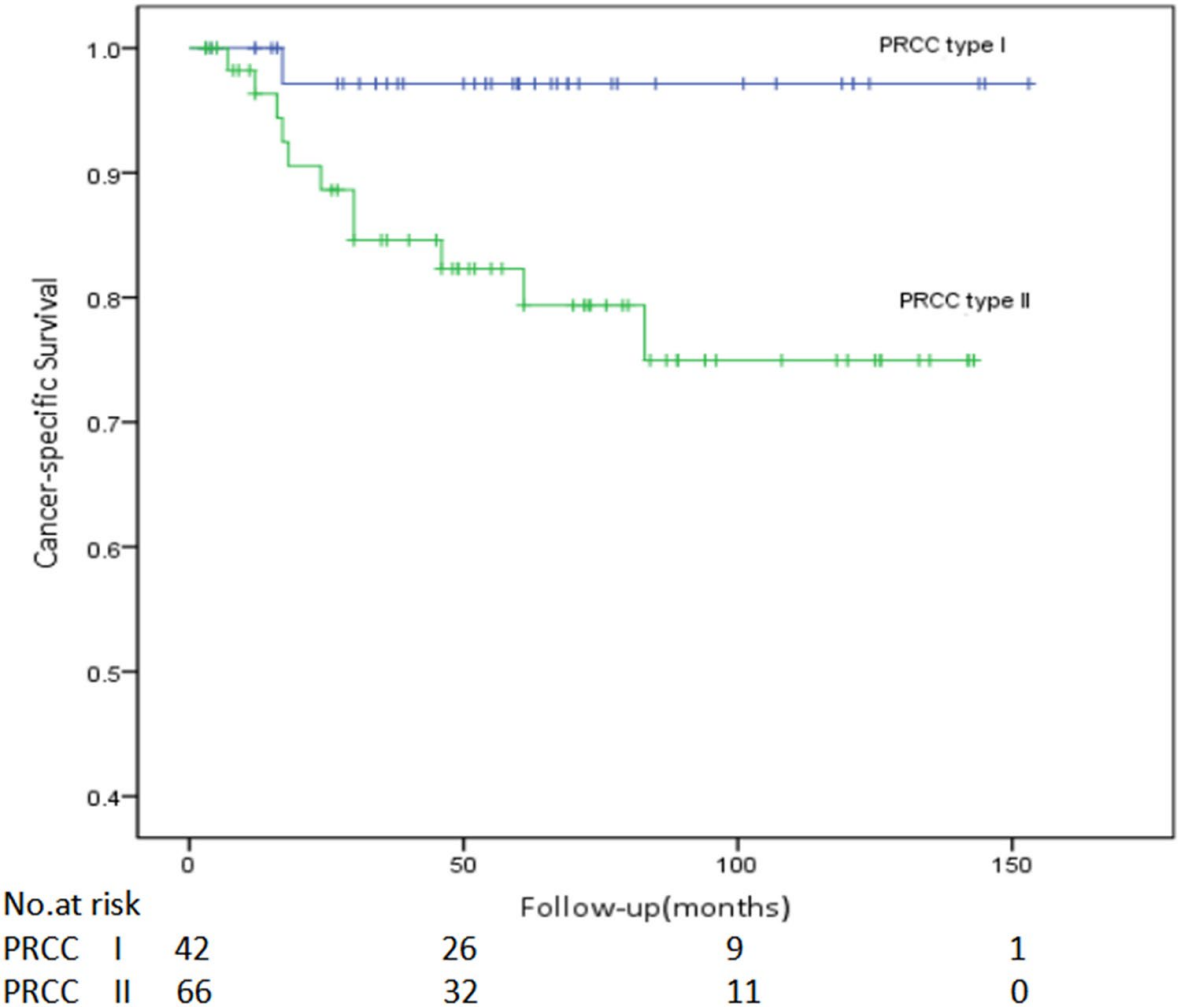
Comparison of cancer-specific survival of type I and type II papillary renal cell carcinoma (PRCC) (*P* = 0.021).

**Figure 2:**
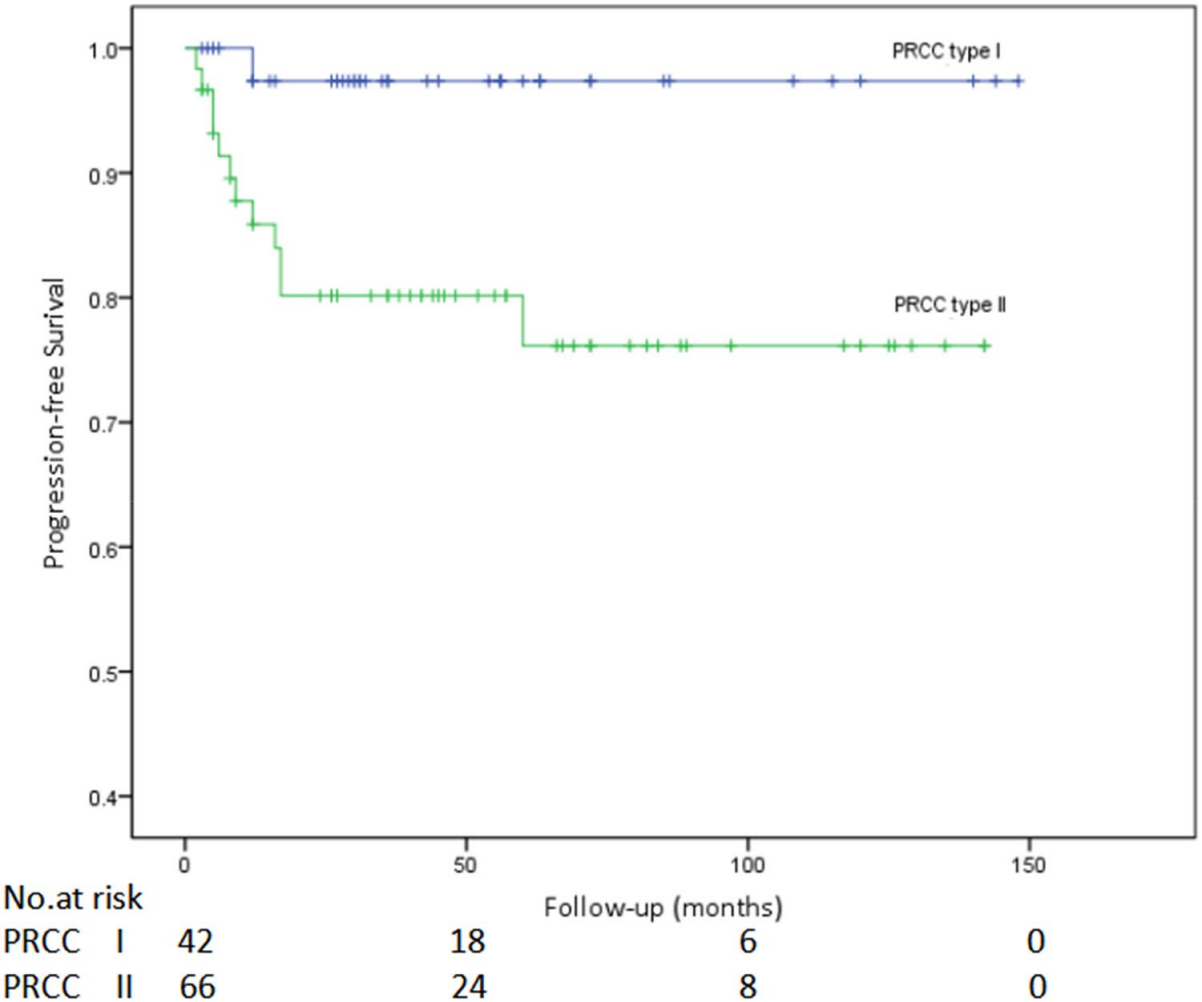
Comparison of progression-free survival of type I and type II papillary renal cell carcinoma (PRCC) (*P* = 0.011).

The Kaplan–Meier survival analysis identified that the cancer-specific survival (CSS) of patients with type I PRCC was significantly better than that of patients with type II PRCC (*P* = 0.021, Fig. 1). The one-year CSS of patients with type I and type II PRCC reached 100% (38/38) and 96.2% (50/52) respectively; while the five-year CSS were 95.2% (20/21) and 75.7% (28/37). Moreover, there was significant difference between the two subtypes in progression-free survival rate (*P* = 0.011, Fig. 2).

Univariate analysis showed that subtypes (*P* = 0.021), symptoms (*P* = 0.024), surgical method (*P* = 0.006), T (*P* < 0.001), N (*P* < 0.001), M (*P* = 0.005),Stage grouping (*P* < 0.001) and WHO/ISUP grading (*P* = 0.025) were associated with survival (Table 2). However, gender (*P* = 0.523) had no significant effect on survival. In a multivariate analysis including the above factors, only Stage grouping was the independent risk factor for prognosis of PRCC patients (*P* = 0.002, Table 2).

**Table 2:** Univariate and multivariate analysis of prognosis of PRCC patients.

Affecting factors	Univariate analysis			Multivariate analysis		
	HR	95%CI	*P*	HR	95%CI	*P*
Gender (male vs female)	1.63	0.36–7.46	0.523	–	–	–
Symptoms (with vs without)	3.62	1.09–12.02	0.024	2.06	0.36–11.70	0.415
Subtypes (II vs I)	7.71	0.99–59.70	0.021	2.07	0.16–26.32	0.573
T (T3 vs T1-2)	31.25	9.01–111.11	< 0.001	1.89	0.20–16.78	0.594
N (N1 vs N0)	17.50	4.94–62.02	< 0.001	2.08	0.28–15.51	0.477
M (M1 vs M0)	7.04	1.45–34.48	0.005	2.14	0.23–19.69	0.502
Stage grouping (III–IV vs I–II)	66.67	13.89–333.33	< 0.001	90.91	5.35–999.98	0.002
WHO/ISUP grading (3–4 vs 1–2)	3.44	1.09–10.87	0.025	1.70	0.39–7.52	0.482
Surgical method (RN vs NSS)	6.45	1.41–29.41	0.006	1.41	0.21–9.71	0.725

**Figure 3:**
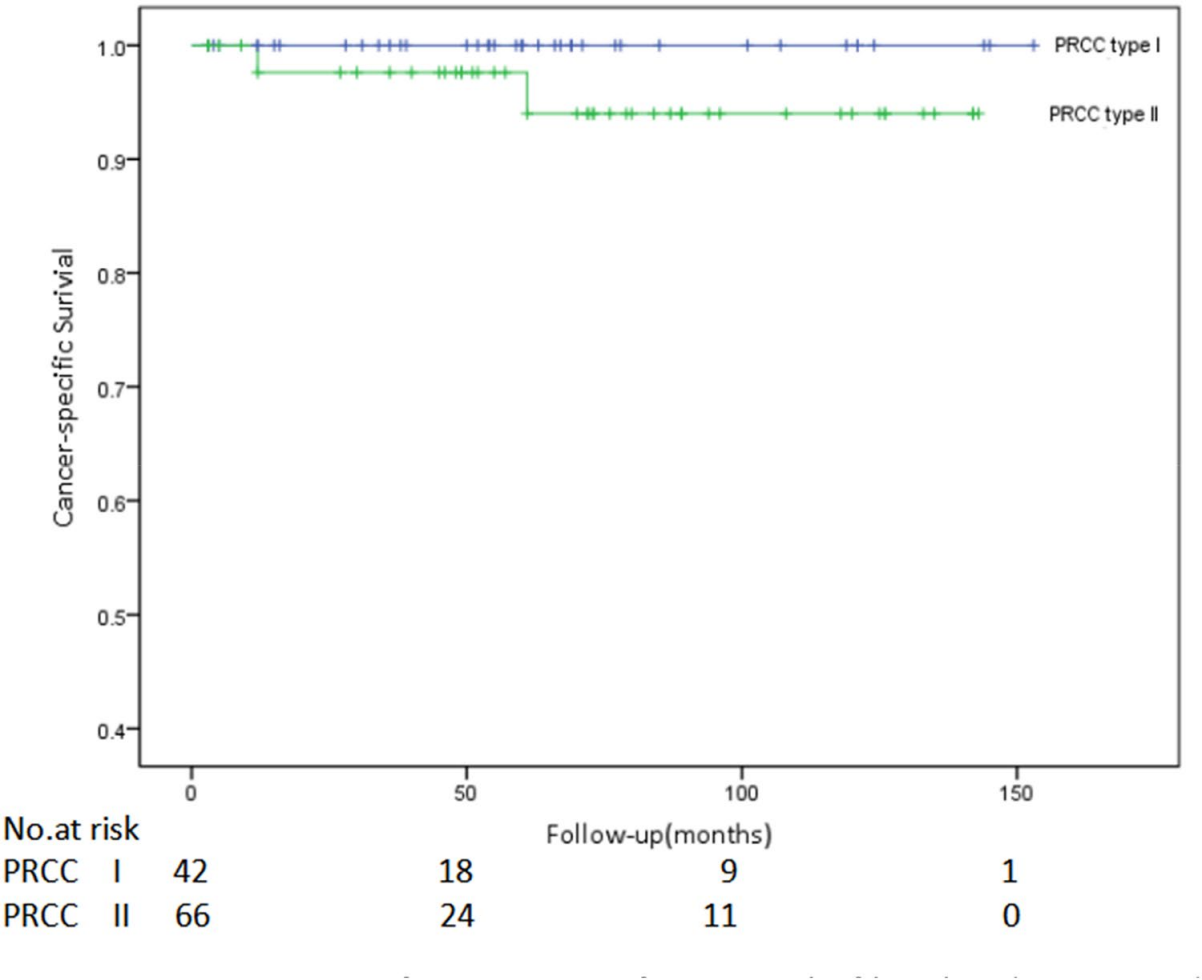
Comparison of cancer-specific survival of localized type I and type II papillary renal cell carcinoma (PRCC) (*P* = 0.214).

**Figure 4:**
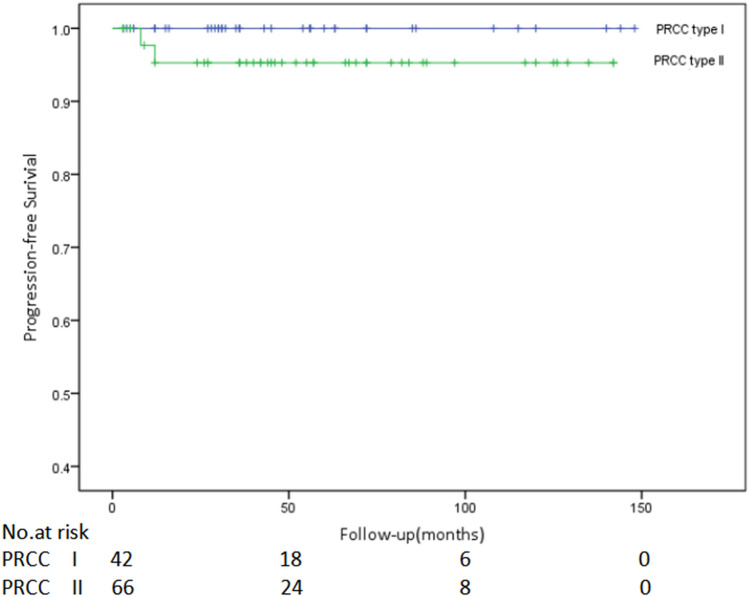
Comparison of progression-free survival of localized type I and type II papillary renal cell carcinoma (PRCC) (*P* = 0.190).

In order to avoid the prognostic effect of Stage grouping, a subgroup survival analysis study on 86 patients with localized PRCC (Stage grouping: stage I–II) was further performed, which included 40 cases of type I patients (35 cases in stage I while 5 in stage II) and 46 cases of type II (42 cases in stage I while 4 in stage II). At the end of follow-up, no tumor progression (including recurrence, metastasis, and death) was found in type I patients and 2 of the type II patients progressed and died. Nonetheless, there were no statistical differences between the two subtypes in cancer-specific survival rate (*P* = 0.214, Fig. 3) and progression-free survival rate (*P* = 0.190, Fig. 4).

## Discussion

PRCC is a well-recognized morphologic variant of RCC. It is characterized by the presence of a papillary or tubule-papillary architecture, and is with neoplastic cells overlying a delicate fibrovascular core or forming compact tubules^2^. While the discrimination between type I and II PRCC is clear at a pathological level, whether this distinction is of clinical value has not been reported.

It has been traditionally thought that PRCC is associated with favorable survival outcomes when compared with conventional ccRCC. The view has been supported by a long-term follow-up study of 607 cases of PRCC patients and 2726 cases of ccRCC patients, which showed that PRCC was associated with better 10-year PFS (92% vs. 67%) and CSS (88% vs. 76%) compared to ccRCC^7^. However, it is not in line with a few of recent studies. For example, Simone et al.^8^ has found that in comparison with ccRCC, PRCC type II is more frequently presented as a disease being either locally advanced or metastatic, which discloses PRCC type II histology as an independent predictor of worse oncologic outcomes. Obviously, PRCC subtype is important for both clinicians and patients since it is associated with prognosis.

It was reported that PRCC accounted for about 10–17% of renal cell carcinoma in Europe and America^5,7,9,10^ , in which most of the patients were classified as Type I^5,6,11^. However, a multi-center data^12^ obtained from Korean patients showed that PRCC only accounted for 5.7% of renal cell carcinoma and was with most type II patients^4^. The clinical features of PRCC appeared to be different in Asia than in Europe and America. In this cohort study with Chinese patients, PRCC accounted for only 5.4% (102/1889). Besides, type II PRCC was dominant (60.7%) in this study. Regarding onset of symptoms, PRCC in the present study was mainly asymptomatic, which was just in common with other renal tumors. According to Steffens et al.^9^ research, the percentage of PRCC patients with initial symptoms was only 24.2%. In contrast, patients presented symptoms in our study group accounted for 35.3%, which may partly result from the dominance of type II PRCC and the high proportion of advanced patients in the present study of Chinese patients. Concerning lesion size, Delahunt et al. proposed no significant difference between type I and II PRCC^13^, which was in accordance with this study. Our study demonstrated that type II patients were characterized by higher T stage, N stage, Stage grouping, WHO/ISUP grading compared with type I patients^13^. Regarding M stage, no further differences are found between the two groups, which is consistent with previous studies^5,11^.

Earlier studies has revealed that the two types of PRCC had obvious differences in imaging and prognosis^2,3^. Delahunt and Eble^2^ found that type II tumors were associated with a higher stage than type I, which accounted for worse prognosis in type II. Geraldine^14^ also found that type II was an independent predictor of cancer-specific mortality. In this study, we found that type II tumors had a higher WHO/ISUP grading (*P* < 0.001) than type I. However, there were no statistically differences in the prognosis between the higher WHO/ISUP grading and the lower. Our study showed that the type I and the type II PRCC were different in both CSS (*P* = 0.021) and PFS (*P* = 0.011) in Kaplan–Meier survival analysis. Meanwhile, we observed that type II was more likely to relapse, which was consistent with the research findings from Paparel et al.^15^, who noted papillary type II accounts for up to 20% of the surgically resected local recurrences.

However, recent multivariate analysis indicated that the subtype was not the independent risk factor affecting prognosis of patients^5,6,11^, which was consistent with our results. Our multivariate analysis revealed that Stage grouping was the only independent risk factor for PRCC prognosis after clinicopathological features were adjusted. At the same time, in order to avoid the influence of Stage grouping as an independent risk factor for prognosis, this study compared CSS and PFS of the two subtypes in localized PRCC (Stage I-II). It further demonstrated that both type I and type II similarly have good prognosis. Ledezma^6^ also revealed no obvious difference in CSS, RFS and OS between the two subtypes in the large sample study comparing 373 cases of type I and 253 cases of type II. Moreover, a multicenter study by Bigot^5^ found that the two PRCC subtypes would not affect the prognosis of patients after following up 369 type I patients and 117 type II patients who had underwent nephron sparing surgery. Futhermore, a recent study with the largest size of metastatic PRCC^16^ demonstrated that type I was similar to type II in PFS and ORR. In summary, these findings suggested that type II PRCC would have more aggressive tumor characteristics than type I, but the two subtypes seemed to have similar oncologic outcomes in both the localized and the metastatic.

With comprehensive molecular characterization of primary PRCC, a recent study revealed that the types I and the type II are different types of renal cancer characterized by specific genetic alterations. The type II may be further classified into three individual subgroups on the basis of molecular differences associated with patient survival^17^. Thus, PRCCs, especially type II, are heterogeneous. The refined subtypes might help improve the prognostic judgment.

The limitations of our study were inherent to its retrospective and single-center study with a limited sample size. The multi-center study with large samples would be conducted in the future studies, which would further confirm the conclusions. Additionally, 5 patients for whom complete pathologic information was not available were excluded, which could have led to a selection bias. The lack of comparison with high Stage grouping (III–IV) between the two groups is an additional limitation of the present study.

In summary, compared with type I PRCC, type II had higher pathological T, N stage and WHO/ISUP grading. However, it was the Stage grouping that made a great difference to oncological outcomes, rather than the subtype of PRCC.

## Methods

The study was conducted in Fujian Provincial Hospital in accordance with the guidelines of the National Institutes of Health of China. All the experimental protocols were approved by the ethics committee affiliated with Fujian Provincial Hospital. All the participants signed the informed consent.

### Clinical data

From January 2008 to September 2020, a total of 107 Chinese with PRCC in Fujian Provincial Hospital and the Jinshan Branch of Fujian Provincial hospital were included in the present study. Five patients could not be further classified because of the lack of pathological specimens. Of the 102 cases definite in classification, 42 were type I PRCC and 60 were type II. There were 99 cases of surgical pathology and 3 cases of biopsy pathology. Among 102 cases, 98 cases were defined using WHO/ISUP grading.

### Pathological diagnosis and staging

The renal carcinoma was graded by using the WHO/ISUP grading system (2016)^18^. Furthermore, postoperative staging was performed according to the 2017 TNM classification developed by the International Union Against Cancer^19^.

### Treatment and follow-up

Radical nephrectomy or nephron sparing surgery was performed based on patients’ willing and the state of tumors. Surgical methods included open surgery and laparoscopic surgery. In addition, ultrasound-guided radiofrequency ablation was applied to two patients due to their actual situations including advanced age, multiple underlying diseases and inability to tolerate surgery. Whether to perform lymph node dissection or not was depended on preoperative imaging and intraoperative exploration results.

### Statistical processing

All data were statistically analyzed by using SPSS 21.0 software. Enumeration data were calculated by Fisher's Exact Probability Method, Pearson χ2 and Wilcoxon Test Method. Measurement data were pre-verified by K-S test and analyzed by applying the Levene Test. Two-sample T test would be employed if conditions were met, in which the measurement data of normal distribution were expressed as $\overline{x}$ ± S, while that of skewed distribution were analyzed by Mann–Whitney U test and indicated by the median and interquartile range. Test standard (α) for the foregoing data was 0.05. The single factor survival analysis data were calculated by using Kaplan–Meier method. In the single factor results, *P* < 0.05 was adopted as the standard into the Cox regression model to conduct multivariate prognostic analysis.
